# Using the Hounsfield Units Derived From Computed Tomography (CT) Scans to Differentiate Between the Subtypes of Allergic Fungal Rhinosinusitis: A Retrospective Study

**DOI:** 10.7759/cureus.80151

**Published:** 2025-03-06

**Authors:** Seham Alsalem, Mohammed Alsalem, Ibrahim Al harithi, Read Al Shehri, Anas Al Zahrani, Muteb Al Khedaidi

**Affiliations:** 1 Radiology, Prince Sultan Military Medical City, Riyadh, SAU; 2 Radiology, King Saud Medical City, Riyadh, SAU; 3 Diagnostic Radiology, King Saud University Medical City, Riyadh, SAU; 4 Radiology, Najran Armed Forces Hospital, Najran, SAU; 5 Radiology, Prince Sultan Medical City, Riyadh, SAU

**Keywords:** allergic fungal mucin, allergic fungal sinusitis, computed tomography scan, diagnosis, hounsfield units, nonfungal sinus opacities, sinus fungal balls

## Abstract

Background

Allergic fungal sinusitis (AFS) is an inflammatory condition, often diagnosed using computed tomography (CT) scans, where Hounsfield units (HU) serve as a critical metric. However, the diagnostic process can be challenging due to the ambiguous patterns of sinus secretions. This study evaluates whether the HU measurements from preoperative CT scans can reliably differentiate between the subtypes of AFS and other chronic rhinosinusitis (CRS) entities by correlating these values with histopathological findings.

Patients and methods

A retrospective analysis was conducted on 120 patients with suspected AFS. All patients had undergone surgical endoscopy at the King Saud Medical City, Riyadh, Saudi Arabia, between 2012 and 2022. Radiographic data, including average, maximum, minimum, and standard deviation (SD) of HU values from unenhanced CT scans, were collected and analyzed. We assessed the diagnostic utility of HU metrics using one-way analysis of variance (ANOVA) and receiver operating characteristic (ROC) curve analysis to determine optimal HU thresholds for differentiating sinus opacities.

Results

Histopathological analysis revealed that 29 (24.2%) cases exhibited non-fungal sinus opacities, 50 (41.7%) displayed sinus fungal balls, and 41 (34.2%) showed allergic fungal mucin. Notably, allergic fungal mucin demonstrated lower heterogeneity and density compared to the other pathologies. Post hoc analysis indicated significant differences in HU maximum values for fungal balls, along with HU average and HU SD values for allergic fungal mucin. ROC curve analysis for fungal balls yielded a high area under the curve (AUC) for HU maximum (AUC=0.868; 95% CI: 0.794-0.923). The optimal HU maximum threshold of 299 provided a sensitivity of 100% and specificity of 71.43% for detecting fungal balls. Allergic fungal mucin showed high AUC values for HU average (AUC=0.979; 95% CI: 0.934-0.996) and HU SD (AUC=0.973; 95% CI: 0.926-0.994). The optimal HU average and HU SD thresholds of 44.0 and 55.6 yielded sensitivities of 90.2% and 100%, and specificities of 100% and 77.1%, respectively.

Conclusion

This study identifies significant correlations between the HU parameters from paranasal CT scans and the pathological features in AFS. Notably, the HU SD and average values correlate with allergic fungal mucin, while HU maximum value indicates the presence of fungal balls. These results suggest that quantitative CT density assessment can aid in differentiating the pathologies of rhinosinusitis. However, external validation is required, and future studies should focus on diverse populations and establish cut-off points for tailored treatment strategies in suspected fungal sinus disease.

## Introduction

Fungal rhinosinusitis presents a complex array of conditions that can be classified as either invasive or noninvasive [1]. Invasive forms include acute, chronic, or granulomatous diseases, whereas noninvasive manifestations may involve localized colonization, fungal balls (mycetoma), and eosinophil-associated fungal rhinosinusitis, with allergic fungal sinusitis (AFS) being a prominent variant [2,3]. AFS predominantly affects immunocompetent individuals with atopy, especially those residing in warm, humid climates. This condition constitutes approximately 5% to 10% of chronic rhinosinusitis (CRS) cases and accounts for up to 32% of patients undergoing sinus surgery, with its prevalence varying across sexes [4,5].

AFS is characterized by hypersensitivity reactions and is associated with elevated total serum immunoglobulin E (IgE) levels along with evidence of inhaled atopy, nasal polyposis, eosinophilic mucus, and positive fungal stains or cultures [4,6]. Despite its increasing recognition, AFS often remains an underdiagnosed clinical entity. Accurate diagnosis is crucial for effective management and typically relies on the integration of clinical features, radiological imaging (particularly CT and MRI), histopathological findings, and immunological evaluations [7,8].

Numerous studies have explored the utility of objective sinus opacity measurements, particularly focusing on Hounsfield units (HU), as potential diagnostic indicators for AFS [9-14]. However, diagnosing it poses challenges due to the variable density signals observed in sinus secretions. Furthermore, the differences in HU values between AFS and other inflammatory sinus diseases remain incompletely understood [10,15]. This study aims to evaluate whether HU measurements from preoperative CT scans can reliably differentiate between the subtypes of AFS and other entities of CRS by correlating these values with histopathological findings. We assessed the diagnostic utility of HU metrics using one-way analysis of variance (ANOVA) and receiver operating characteristic (ROC) curve analysis to determine the optimal HU thresholds for differentiating sinus opacities. Unlike previous studies, which primarily focused on qualitative assessments of sinus opacities, this study establishes precise quantitative HU thresholds for distinguishing between fungal and nonfungal sinus disease. Given the clinical importance of differentiating the AFS subtypes, we seek to address this question: Can the degree of sinus opacity, measured by HU, be used to differentiate between AFS and other forms of CRS?

## Materials and methods

Study design and setting

This retrospective study analyzed data from 120 patients with suspected AFS, who had undergone endoscopic sinus surgery (ESS) at King Saud Medical City, Riyadh, Saudi Arabia, between 2012 and 2022. The study protocol conformed to the ethical principles of the Declaration of Helsinki and received ethical approval from the Institutional Review Board (IRB) of King Saud Medical City (ID: H-01-R-053). Following a detailed explanation of the study objectives, informed consent was obtained from all participants, authorizing the extraction of data from their medical records and the publication of their anonymized medical information and images.

Inclusion and exclusion criteria

The inclusion criteria consisted of adult patients (aged over 15 years) who had undergone ESS and had preoperative CT scans conducted within 30 days before surgery. Additionally, patients were required to have postoperative microbiological or histopathological results available. Patients were excluded from the study if they had undergone ESS for sinonasal malignancies, lacked definitive pathological confirmation of their condition, or did not have perioperative CT scans. Additionally, patients with active sinus infections were excluded to minimize variability in sinus tissue density measurements. Individuals who had undergone related surgeries within the past year were also excluded to ensure a more homogeneous study population. 

Calculation of sample size

To determine the sample size for our study, we utilized parameters derived from the findings of Killeen et al. [10], who reported significant area under the curve (AUC) values for the detection of fungal balls and allergic fungal mucin. For fungal ball detection, we assumed a sensitivity of 72.7%, while for allergic fungal mucin, a sensitivity of 100% was considered. We applied the formula for calculating sample size for two proportions, establishing a confidence level of 95% (Zα/2 = 1.96) and a power of 80% (Zβ = 0.84). Using the calculated proportions of p₁=0.727 and p₂=1.0, the initial sample size estimation yielded approximately 21 participants per group. To account for an anticipated dropout rate of 10%, the adjusted sample size was recalculated to be approximately 24 participants per group.

CT scan protocol

Sinus CT scans were evaluated using the Sante DICOM and Communications in Medicine viewer (Santesoft LTD, Cyprus), employing the elliptical selection tool to quantify soft tissue densities within the sinuses [10]. The scans were acquired using a Siemens SOMATOM Definition Flash (Siemens Healthineers, Germany) with a slice thickness of 2 mm and exposure settings of 120 kV and 200 mA. The largest and most representative region of interest within each sinus opacity was analyzed, carefully excluding air pockets, "frothy" areas, as well as osseous and dental structures specified in previous studies [9,10]. For the analysis of bone density, gray values (GVs) from the 3D CT scans were quantified as HU, with the values calculated during image reconstruction based on the materials present in each voxel. Preprocessing steps included the application of noise reduction algorithms and standardization procedures to ensure the accuracy of the HU measurements. The radiographic densities assessed included the maximum HU (HU max), minimum HU (HU min), average HU (HU avg), and standard deviation of HU (HU SD). All radiographic data were collected and analyzed by blinded reviewers prior to the examination of patient medical records.

Data extraction, collection, and measurement

Strict data collection protocols were adhered to for ensuring data quality and consistency from multiple medical records. This included the development and implementation of a comprehensive checklist which focused on patient demographics, relevant clinical characteristics, radiographic findings, and pathological information. Quality assurance measures included rigorous training for data abstractors, with instructions to cross-validate each entry against the original medical records. Regular audits were conducted to identify discrepancies and ensure adherence to the checklist criteria. Any data inconsistencies were promptly addressed through a re-evaluation of the records by a supervising team of healthcare professionals. Comprehensive data collection encompassed age, gender, presenting symptoms, HU parameters, and definitive pathological diagnoses. Radiographic images were independently reviewed by two radiologist authors who were blinded to the clinical data of the patients. Blinding was enforced by using anonymized datasets, ensuring that the reviewers were unable to access any patient history or identifiable information. Interobserver reliability was assessed using weighted Cohen’s kappa and intraclass correlation coefficient (ICC) analyses.

Main outcome

The primary objective of this study was to assess the diagnostic accuracy of various HU values derived from CT scans in differentiating the subtypes of AFS.

Statistical analysis

Descriptive statistics, including means and SDs, summarized quantitative data, while frequencies and percentages addressed categorical variables. The Kolmogorov-Smirnov and Levene tests examined the normality and homogeneity of variance, respectively. The reliability of HU measurements among radiologists was assessed using kappa statistics and the ICC to evaluate agreement among raters. Radiographic densities of the sinus opacities on the CT scans (HU avg, HU max, HU min, and HU SD) were analyzed using one-way ANOVA, and based on the histopathological findings were categorized into three groups: nonfungal sinus opacities, sinus fungal balls, and allergic fungal mucin. The predictive performance of these radiographic characteristics was evaluated using the Youden index, derived from ROC curves, which provided insights into the AUC, specificity, sensitivity, and accuracy. A p-value of <0.05 was deemed statistically significant. All analyses were conducted using IBM SPSS Statistics for Windows, Version 22 (Released 2013; IBM Corp., Armonk, New York, United States).

## Results

Patient characteristics

**Figure 1:**
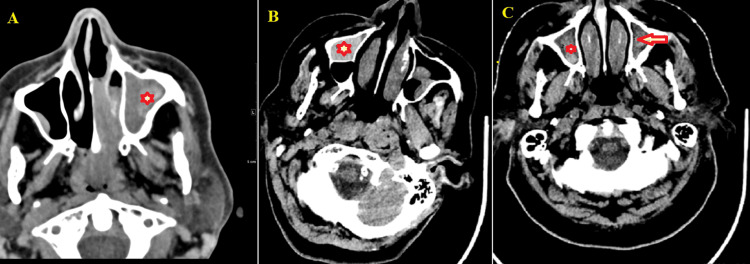
Non-enhanced computed tomography (CT) scans of the paranasal sinuses and the brain (A) Non-enhanced computed tomography (CT) scan of the paranasal sinuses with a soft tissue window demonstrates complete inhomogeneous opacification of the left maxillary sinus (asterisk). Within this sinus, the hyperdense material with an average density of 85 Hounsfield units (HU) is centrally located and surrounded by hypodense mucosa. This finding was pathologically proven to be allergic fungal sinusitis. (B) Non-enhanced CT scan of the brain demonstrates bilateral maxillary sinus retention cysts. The right maxillary sinus (asterisk) displays a hyper-attenuated retention cyst with an average density of 58 HU. (C) Non-enhanced CT scan of the brain reveals bilateral maxillary sinus mucosal thickening, with hyperdense materials exhibiting an average density of 37 HU in the left maxillary sinus (arrow) and 35 HU in the right maxillary sinus (asterisk). These findings are indicative of chronic sinusitis with inspissated secretion.

This study included 120 patients with a mean age of 32.9±15.4 years (range: 18.0-72.0 years). The majority were male (n=81, 67.5%) and the most common symptoms were rhinorrhea (n=90; 75.0%), followed by headache (n=69; 57.5%) and postnasal drip (n=60; 50.0%). Histopathological analysis showed nonfungal sinus opacities in 29 (24.2%), fungal balls in 50 (41.7%), and allergic fungal mucin in 41 (34.2%) patients (Figure 1A-1C; Table 1).

**Table 1:** Characteristics of patients with fungal sinus disease SD: standard deviation. *Some patients had multiple symptoms.

Variables	N (%)
Age (years), mean ±SD	32.9 ± 15.4 (range 18.0-72.0)
Gender	
Male	81 (67.5%)
Female	39 (32.5%)
Number of sinuses involved	
One	42 (35.0%)
Two	60 (50.0%)
Three or more	18 (15.0%)
Symptoms*	
Rhinorrhea	90 (75.0%)
Headache	69 (57.5%)
Postnasal drip	60 (50.0%)
Obstruction	30 (25.0%)
Hyposmia	21 (17.5%)
Involved sinus	
Maxillary	33 (84.6%)
Sphenoid	26 (66.7%)
Ethmoidal	13 (33.3%)
Histopathological type	
Fungal ball	50 (41.7%)
Allergic fungal mucin	41 (34.2%)
Nonfungal opacities	29 (24.2%)

Quantitative radiographic density attributes of the sinus opacities

The findings derived from CT scans elucidated significant discrepancies in the HU measurements across various histopathological subtypes of sinusitis. Notably, fungal balls demonstrated the highest HU max (378.59±43.93), reflecting a markedly elevated radiological density when compared to allergic fungal mucin (218.98±32.70) and nonfungal opacities (190.38±47.90), with p-values corroborating their statistical significance (p<0.001). Furthermore, the HU avg associated with allergic fungal mucin revealed a substantial decrease relative to fungal balls (-21.94) and nonfungal opacities (52.47), highlighting the distinctive radiological profiles among these subtypes. Although HU min values did not exhibit statistical significance, the higher HU SD observed in fungal balls (63.69±7.23) indicates a greater variability in density within this category. Collectively, these findings underscore the integral role of HU measurements in differentiating histopathological types of sinusitis, thereby facilitating precise diagnosis and informed treatment strategies, as further illustrated in Table 2. 

**Table 2:** HU measurements and their correlation with histopathological subtypes of sinusitis HU: Hounsfield units, SD: standard deviation, MD: Mean difference. ^*^One-way ANOVA (p-values of <0.05 were considered significant). ^†^Post hoc tests (p-values of <0.05 were considered significant). ^‡^Mean difference between fungal balls and allergic fungal mucin. ^§^Mean difference between fungal balls and nonfungal opacities. ^¶^Mean difference between allergic fungal mucin and nonfungal opacities.

Radiologic sinus opacities	Type	Mean ± SD	p-value^*^	MD	p-value^†^
Maximum HU	Fungal ball	378.59 ± 43.93		—	—
	Allergic fungal mucin	218.98 ±32.70		159.61^‡^	
	Nonfungal opacities	190.38 ±47.90		188.21^§^	
				28.60^¶^	0.008
Average HU	Fungal ball	55.95 ±12.21	<0 .001	—	—
	Allergic fungal mucin	77.88 ±12.89		-21.94^‡^	
	Nonfungal opacities	25.41±9.47		30.53^§^	
				52.47^¶^	<0.001
Minimum HU	Fungal ball	3.28 ±1.82	0.219	—	—
	Allergic fungal mucin	3.00 ±1.79		0.28^‡^	0.714
	Nonfungal opacities	2.62 ±1.32		0.66^§^	0.256
				0.38^¶^	0.602
SD of HU	Fungal ball	63.69 ±7.23		—	—
	Allergic fungal mucin	42.79 ±3.88		20.90^‡^	
	Nonfungal opacities	30.48 ±16.17		33.21^§^	
				12.31^¶^	

The reliability of HU measurements among radiologists was assessed using kappa statistics, resulting in a kappa value of 0.75 (p<0.05), indicating substantial agreement. The ICC was also 0.80, further supporting the consistency of measurement.

Predicting fungal sinus disease using radiographic density

*Fungal Balls* 

**Figure 2:**
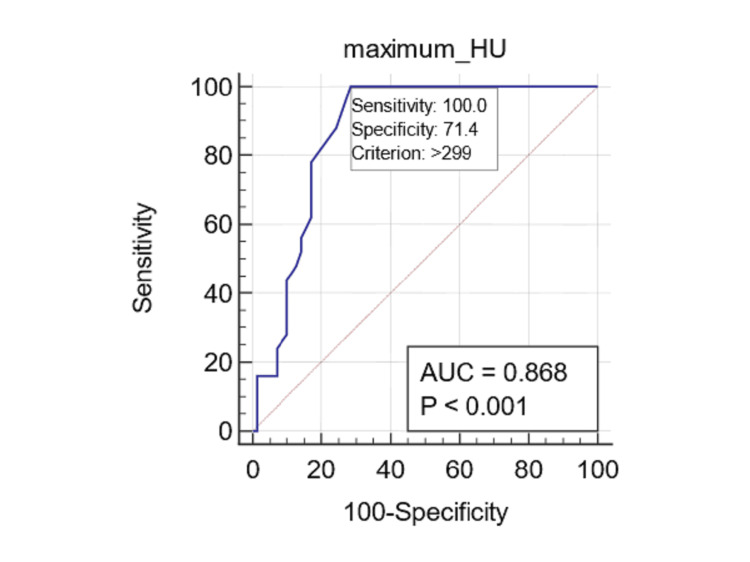
Predicting fungal balls with Hounsfield units (HU) The receiver operating characteristic (ROC) curve for the maximum HU indicates the highest area under the curve (AUC) of 0.868, with a sensitivity of 100% and specificity of 71.4% for predicting fungal balls, using an optimal cutoff of 299 HU.

The ROC curve analysis conducted for fungal balls revealed a notably high AUC for HU max measurements (AUC: 0.868; 95% CI: 0.794 to 0.923; p<0.0001), signifying strong predictive validity. In contrast, alternative measurements, including HU min (AUC: 0.555; 95% CI: 0.462 to 0.646; p=0.299), HU avg (AUC: 0.578; 95% CI: 0.485 to 0.668; p=0.1753), and HU SD (AUC: 0.508; 95% CI: 0.411 to 0.598; p=0.947), exhibited significantly lower levels of accuracy. By employing the Youden index derived from the ROC curves (by maximizing combined sensitivity and specificity), an optimal threshold for HU max at 299 yielded a sensitivity of 100% and a specificity of 71.43% for the detection of fungal balls (Figure 2).

Allergic Fungal Mucin

**Figure 3:**
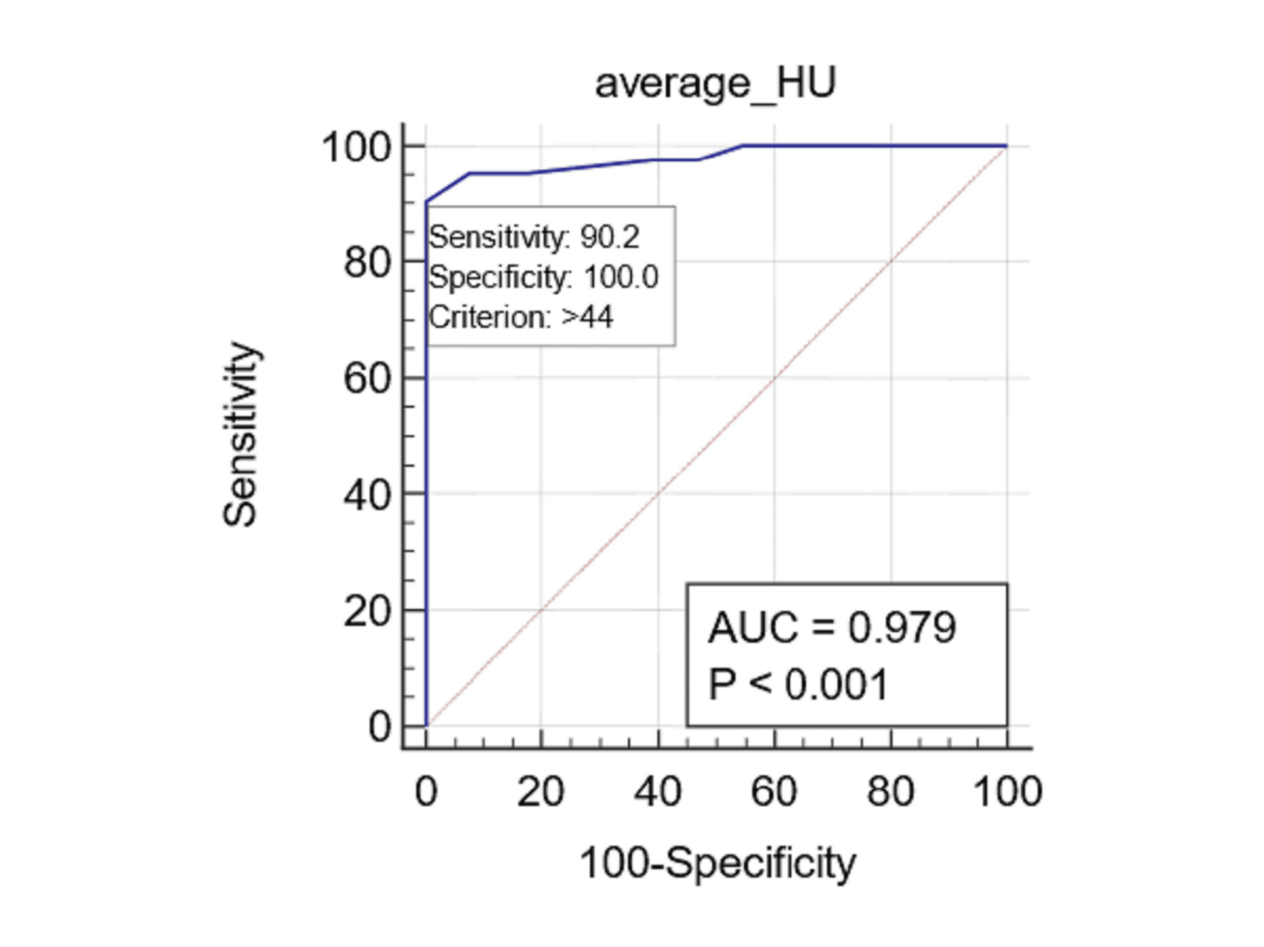
Predicting allergic fungal mucin with Hounsfield units (HU) The receiver operating characteristic (ROC) for the average HU (HU avg) predicts the presence of allergic fungal mucin, achieving an accuracy of 0.979, a sensitivity of 90%, and a specificity of 100% at a threshold of 44 HU.

**Figure 4:**
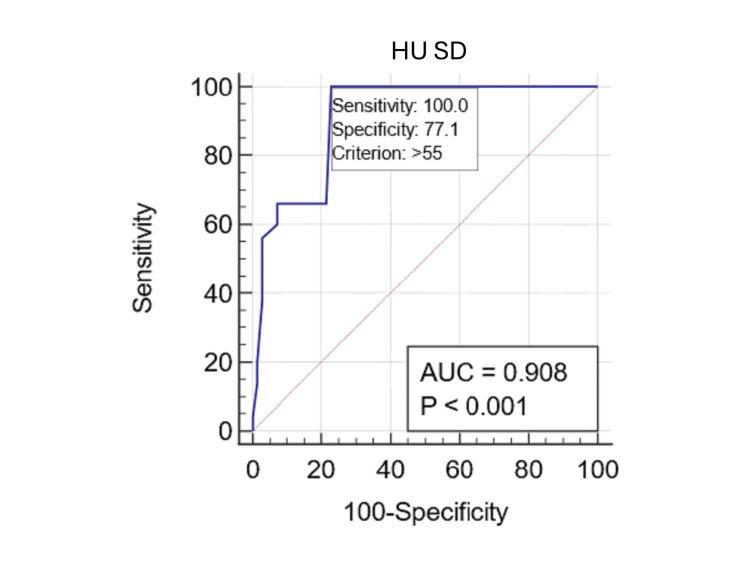
Predicting allergic fungal mucin with standard deviation of Hounsfield units (HU) The receiver operating characteristic (ROC) curve for the standard deviation of HU (HU SD) indicates allergic fungal mucin presence, yielding an accuracy of 0.908, with a sensitivity of 100% and a specificity of 77.1% at a cutoff of 55 HU.

The ROC curve analysis pertaining to allergic fungal mucin indicated an exceptionally high AUC for HU avg (AUC: 0.979; 95% CI: 0.934 to 0.996; p<0.0001) (Figure 3) and HU SD (AUC: 0.973; 95% CI: 0.926 to 0.994; p<0.0001) (Figure 4).

Conversely, HU max (AUC: 0.505; 95% CI: 0.412 to 0.597; p=0.9271) and HU min (AUC: 0.504; 95% CI: 0.411 to 0.597; p=0.936) values did not reveal statistically significant differences. By applying the Youden index (by maximizing combined sensitivity and specificity), a criterion value of 44.0 for HU avg yielded a sensitivity of 90.2% and a specificity of 100% for detecting allergic fungal mucin. Furthermore, a criterion of 55.6 for HU SD resulted in a sensitivity of 100% and a specificity of 77.1% for their identification. 

## Discussion

This study investigated the reliability of HU measurements from preoperative CT scans in differentiating between the subtypes of AFS and other entities of CRS. These values were then correlated with histopathological findings while utilizing a larger sample size than previous studies to enhance the robustness of the statistical analyses. Our results demonstrated that the HU SD and HU avg parameters from paranasal CT scans were significantly correlated with the presence of allergic fungal mucin. Additionally, the HU max parameter was strongly associated with fungal balls, establishing new threshold values for improved diagnostic accuracy. These findings help advance the field of radiologic evaluation for AFS and provide clearer guidance to clinicians for distinguishing between AFS subtypes and other CRS entities.

The conventional diagnosis of noninvasive fungal sinusitis often relies on subjective interpretations of CT scans. While high-density opacities within the sinuses suggest fungal involvement, these findings must be integrated with other clinical indicators. For instance, the presence of hyperattenuating foci, nasal polyps, and elevated serum anti-Aspergillus IgE levels have been shown to predict AFS with sensitivity of 70% and specificity of 100% [16,17]. Furthermore, sinus fungal balls typically present high-density opacities, often accompanied by calcifications, density variation (heterogeneity), and sinus wall erosion. CT imaging can identify fungal balls with sensitivities between 62% and 83% and specificities ranging from 94% to 99% [16,18]. A significant limitation in existing research is the reliance on subjective assessments rather than objective, measurable criteria.

CRS patients exhibit bone remodeling characterized by increased periosteal thickness, elevated osteoblast and osteoclast activity, and subsequent woven bone formation and marrow fibrosis [19-21]. Advanced bone remodeling grades correlate with increased mucosal grades in histological assessments as well as heightened soft tissue and bone density on CT scans, highlighting the complex pathophysiology of CRS [4,15,22]. Research has linked bone remodeling in CRS to abnormal bony thickening (hyperostosis) of the sinus walls on CT scans [12,22]. However, objective methodologies for grading bone remodeling through CT imaging remain limited, as most staging systems focus on soft tissue evaluations [23]. Our investigation explored the role of HU in evaluating AFS subtypes and correlating them with histopathological findings. It demonstrated significant associations between HU SD and HU avg with allergic fungal mucin diagnosis, while HU max correlated strongly with fungal balls. These results reinforce the utility of HU as standardized measurements in CT evaluations.

High-resolution CT imaging provides valuable information for surgical planning in sinonasal interventions. Characteristic opacifications often define fungal sinus disease [11]. Studies have documented significant variations in HU values across different AFS subtypes, underscoring the potential of CT scans in differentiating them [9,10,24]. For example, Sedaghat et al. found that sinus opacification parameters, particularly HU density, enhance correlations between CT findings and symptom severity in patients with CRS [9]. Killeen et al. demonstrated that features identified in sequential CT scans significantly predict the presence of mycetoma or allergic mucin [10]. Despite these advancements, achieving high sensitivity and specificity remains a challenge, highlighting the need for integrated clinico-radiological evaluation. Our data corroborate previous research linking hyperattenuating opacities and calcifications to allergic fungal mucin and fungal balls. Notably, our study indicated that HU avg effectively predicts allergic fungal mucin, while lower HU SD indicates homogeneity of opacity. These findings are consistent with those of Killeen et al. and Huang et al. [10,25]. In their studies, which employed a similar quantitative CT approach, the authors noted the relatively homogeneous appearance and elevated density of allergic fungal mucin when compared to other CRS conditions [10,25]. This consistency across multiple studies strengthens the argument that these radiographic characteristics hold diagnostic significance in the evaluation of AFS.

Prior studies have indicated that the HUs of sinus opacities in fungal balls are substantially higher than those associated with odontogenic maxillary sinusitis or allergic fungal mucin [10,14,25,26]. This elevation is attributed to dense fungal hyphae and metabolic deposits, contributing to high-density opacities. Our study revealed that HU max was statistically significant in predicting the presence of fungal balls, reinforcing findings reported by other studies [10,25]. A high HU max often suggests dense components within the opacity and is characterized by substantial specificity. In our study, a HU max of 299 demonstrated a sensitivity of 100% and specificity of 71.43% for the detection of fungal balls. In their report, Huang et al. established an optimal HU cutoff of >101.17 for fungal lesions, yielding a sensitivity of 96.6% and specificity of 100% [25]. Furthermore, Killeen et al. identified a maximum HU cutoff of 334.0 for detecting fungal balls with a sensitivity of 90.9% and specificity of 72.7% [10]. Overall, CT imaging using sinus opacification and hyperattenuated areas as diagnostic criteria exhibited a sensitivity of 62% and specificity of 99% [16]. However, diagnosing fungal balls preoperatively remains challenging without visible intralesional calcifications. Cha et al. [27] reported a low sensitivity (74%) for hyperdensity as a singular CT feature, emphasizing the need for clinical indicators such as patient age and additional radiographic features to enhance CT sensitivity in diagnosing fungal balls within the maxillary sinus. Moreover, inadequate mucociliary clearance can trigger inflammatory responses in the sinus mucosa, leading to obstruction of the natural ostium and potential complications, including sinus fullness and secondary bacterial infections [27,28]. These findings underscore the necessity for a multifaceted approach in the preoperative evaluation of suspected fungal balls by integrating both radiographic and clinical parameters to optimize diagnostic accuracy.

The accumulation of allergic fungal mucin leads to distinctive radiographic features of AFS. CT scans typically reveal heterogeneous signal intensity in paranasal sinuses affected by AFS, with manifestations including sinus wall expansion, remodeling, or thinning due to mucin accumulation [10,14,25,26]. It is noteworthy that sinonasal sarcomas or meningiomas may yield similar radiographic findings [29]. Our findings unveiled a distinct correlation between HU SD and HU max, reflecting the heterogeneity of sinus contents when comparing allergic fungal mucin to fungal balls. Specifically, allergic fungal mucin presented with lower heterogeneity than other sinus opacities (e.g., fungal balls and nonfungal opacities). Notably, we identified an HU SD threshold of 55 for allergic fungal mucin and an HU max threshold of 299 for fungal balls. These results echo studies by Killeen et al. and Huang et al. [10,25], reinforcing the value of implementing objective numeric density measures in diagnosing sinus diseases. 

An average opacity density exceeding 44 HU indicated the presence of allergic fungal mucin, achieving 90% sensitivity and 100% specificity. This supports earlier findings linking hyperattenuating sinus opacities and calcifications in fungal balls [10,25]. Our study demonstrates that elevated HU avg values in sinus opacities effectively differentiate fungal balls from allergic fungal mucin. Killeen et al. reported a 42.9 HU cutoff with 100% sensitivity and 46.3% specificity, which increased to 73.2% with the addition of nasal polyposis [10]. Tunç et al. found that a HU max cutoff of 241 yielded sensitivity of 84.6% and specificity of 77.3%, while a mean cutoff of 41.5 provided sensitivity of 76.9% and specificity of 86.4% for detecting fungal lesions [14]. Variability in HU values across studies may arise from differences among sinus types and radiation exposure during CT imaging.

Study limitations

This study has several limitations that must be acknowledged. While we utilized objective density metrics, the absence of advanced quantitative assessments may constrain our predictive capabilities regarding fungal sinusitis. Specifically, we did not analyze density histograms from sinus openers, limiting our ability to examine non-normal density distributions relevant to fungal conditions. Additionally, our focus on noninvasive sinus disorders restricts the generalizability of our findings to invasive forms of sinusitis. Importantly, we did not correlate our assessments with symptom severity, which warrants further investigation. The single-center design, combined with a limited sample size and retrospective nature, raises concerns regarding external validity, particularly in relation to varying imaging protocols across different populations and institutions. The reliance on secondary data and variability in the regions of interest selection may also introduce inconsistency. Future research should aim to include larger and more diverse patient cohorts while addressing these limitations to enhance the applicability of our findings across different clinical settings.

## Conclusions

This study highlights significant correlations between HU parameters obtained from paranasal CT scans and specific pathological features in AFS. Notably, a statistically significant association was found between HU SD and HU avg values and allergic fungal mucin, while the HU max value correlated strongly with the presence of fungal balls. These results suggest that quantitative CT density assessment can serve as a valuable adjunctive diagnostic tool for differentiating rhinosinusitis pathologies. However, it is crucial to underscore the necessity for external validation of these findings before clinical implementation. Future studies should aim to replicate these results across diverse populations to ensure their robustness. Establishing specific cut-off points linked to surgical and pathological outcomes may further refine treatment strategies for patients suspected of fungal sinus disease.
